# Photoionization and Photofragmentation Dynamics of I_2_ in Intense Laser Fields: A Velocity-Map Imaging Study

**DOI:** 10.1021/acs.jpca.2c04379

**Published:** 2022-11-09

**Authors:** Felix Allum, Joseph McManus, Oskar Denby, Michael Burt, Mark Brouard

**Affiliations:** Chemistry Research Laboratory, Department of Chemistry, University of Oxford, Oxford OX1 3TA, U.K.

## Abstract

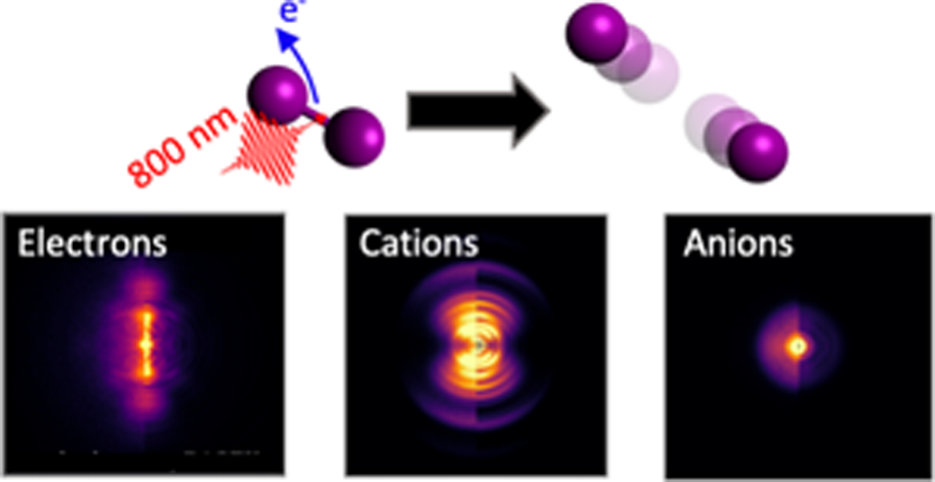

The photoionization and photofragmentation dynamics of I_2_ in intense femtosecond near-infrared laser fields were studied using velocity-map imaging of cations, electrons, and anions. A series of photofragmentation pathways originating from different cationic electronic states were observed following single ionization, leading to I^+^ fragments with distinct kinetic energies, which could not be resolved in previous studies. Photoelectron spectra indicate that these high-lying dissociative states are primarily produced through nonresonant ionization from several molecular orbitals (MO) of the neutral. The photoelectron spectra also show clear signatures of resonant ionization pathways (Freeman resonances) to low-lying bound ionic states via Rydberg states of the neutral moiety. To investigate the role of these Rydberg states further, we imaged anionic products (I^–^) formed through ion-pair dissociations of neutral molecules excited to these Rydberg states by the intense femtosecond laser pulse. Collectively, these results shed significant new light on the complex dynamics of I_2_ molecules in intense laser fields and on the important role of neutral Rydberg states in a full description of strong-field phenomena in molecules.

## Introduction

In recent years, the interaction of molecules with intense femtosecond laser pulses has garnered considerable attention as they form the foundation of a range of experimental techniques that can be used to study ultrafast photochemistry and photophysics. Such techniques include Coulomb explosion imaging,^1−4^ high-harmonic spectroscopy,^5−10^ and laser-induced electron diffraction.^11−14^ A detailed understanding of the dynamics induced by intense fields is crucial to the development of these experimental approaches. However, the photoinduced dynamics of even the simplest molecules in intense laser fields may be very rich and complex,^15^ due to the range of processes which can be driven by the optical field. Such exotic phenomena include field-induced coupling and distortion of potential energy surfaces,^16−18^ charge-resonance enhanced ionization,^19,20^ dynamic and geometric alignment,^21−23^ and multielectron ionization dynamics.^6,7,24−29^

Studies into strong-field ionization of small molecules have demonstrated that ionization from deeper-lying orbitals than the highest-occupied molecular orbital (HOMO) may be significant.^24^ Ionization from these more strongly bound orbitals (HOMO–1, HOMO–2, etc.) leaves the molecular cation in electronically excited states and therefore predetermines, to a large degree, the nuclear dynamics (e.g., photodissociation pathways) following ionization. Such multielectron ionization dynamics were observed some time ago in N_2_ using photoelectron spectroscopy,^24^ but they have since been observed in a series of molecules using various experimental techniques, such as photoelectron (coincidence) imaging,^25−28^ high-harmonic spectroscopy,^6,7,29^ and photoelectron angular streaking.^26^ Of particular interest to the current work is experiments that have established the connection between the ionized orbital and the photofragmentation dynamics observed following ionization. This includes a seminal study by Stolow and co-workers on *n*-butane and 1,3-butadiene using electron–ion covariance spectroscopy.^30^ In this work, by examining the above-threshold ionization (ATI) photoelectron spectra correlated to production of a specific photoion product, the role of ionizing to different ionic continua could be clearly interrogated. Recent work by Weinacht and co-workers,^31^ employing photoion–photoelectron coincidence imaging on the D_2_O molecule could further distinguish different photofragmentation pathways leading to the same cation products with different kinetic energy releases. The above-threshold photoelectron spectra correlated to different photofragmentation pathways encoded information about the ionization pathways in operation and how the ionization channel predetermined the ionic photofragmentation dynamics.

Here we study the dynamics of I_2_ molecules subjected to intense femtosecond laser fields in the near-infrared (NIR) using velocity-map imaging (VMI).^32,33^ As I_2_ consists entirely of heavy atoms, the role of nuclear motion during the laser field is minimized, and thus the photofragmentation dynamics can be expected to be dominated by the electronic states populated in the field. Recent work by Gibson and co-workers studied the strong-field induced fragmentation dynamics of I_2_^34^ also using VMI at a series of laser wavelengths. Distinct features in the kinetic energy distributions of I^+^ fragments produced following single ionization were observed, and they were suggested to occur following excitation of deep-lying molecular orbitals of I_2_. This assignment was strengthened by time-resolved measurements, which suggested that the relatively high velocity I^+^ atoms observed originate from dissociative states populated directly in the laser field, not via population of the bound X and A states of the cation formed by ionization from the HOMO or HOMO–1.

In the present work, measurements with higher velocity resolution allows unambiguous distinction of at least six dissociative ionization channels, once more assigned to population of different electronic states of the I_2_^+^ cation producing fragments with different asymptotic velocities. Evidence for similar behavior underpinning the Coulomb explosion of higher parent charge states is also presented. Further experiments measuring photoelectron velocity distributions at a range of incident intensities are consistent with multiple nonresonant ionization pathways and additionally demonstrate the important role of Freeman resonances^35^ in this system. Assignments of several of the Rydberg states resonantly populated in the laser field are offered. Ion-pair dissociations^36−38^ from these neutral Rydberg states which may remain populated following the light–matter interaction are also studied through the detection of negative I^–^ ions, which also show different photofragmentation pathways. To our knowledge, this is the first report of anion production from molecules in intense laser fields by this mechanism, but it may be expected to be a more general phenomena in (typically halogen containing) molecules with excited states of ion-pair character.^37,39^ Collectively, the results presented provide several new insights into the behavior of small molecules in intense laser fields, and furthermore establish I_2_ as a fertile system for future study into molecular strong-field ionization and photofragmentation dynamics.

## Experimental Methods

The experimental apparatus consists of a velocity-map imaging spectrometer, as has been described in previous work,^40,41^ operating in the conventional velocity-map^33^ imaging^32^ configuration. The I_2_ sample was prepared by diluting the room temperature vapor pressure of solid I_2_ in helium, to a total pressure of 1–2 bar (∼0.02% I_2_). This mixture was introduced into the spectrometer as a pulsed molecular beam, using a Series 9 General Valve. Following collimation through a skimmer, the molecular beam passes through into the spectrometer’s interaction region. The fundamental pulsed output (800 nm, ∼40 fs duration) of a commercial Ti:sapphire amplifier (Spectra-Physics Solstice Ace) was used to initiate ionization. The pulses were variably attenuated by adjusting the angle of a λ/2 waveplate installed prior to a thin film polarizer. The transmitted beam was then focused by a 200 mm focal length lens into the interaction region of the spectrometer, perpendicularly intersecting the molecular beam. The peak intensity at the focus was varied in the range 30–120 TW cm^–2^.

Nascent charged particles were accelerated by velocity-mapping fields to a time- and position-sensitive detector consisting of dual stacked microchannel plates (MCPs) coupled to a P47 phosphor screen. For the data presented in this work, the spectrometer was either operated in a positive potential mode to image positively charged ions or in a negative potential mode to image electrons and anions. In both cases, voltages were applied to the repeller and extractor electrodes in a 1:0.886 ratio, with other electrodes held at ground. For the majority of the data presented in the manuscript, an absolute voltage of 8 kV was applied to the repeller, while to record a high quality image of the low velocity I^–^ ions discussed later in the manuscript, the repeller voltage was lowered to −1 kV. The impact of each accelerated particle at the detector produced a flash of light, which was imaged by a fast timestamping camera, the Pixel Imaging Mass Spectrometry (PImMS2) camera.^42,43^ This allowed the time and position of each event to be determined, enabling all particles of a given charge to be imaged in a single acquisition, with the timing of the event indicative of the particle’s mass-to-charge ratio. Images were acquired over tens of thousands of laser cycles. The experiment was operated at 10 Hz, due to limitations on the repetition rate at which the PImMS2 camera can acquire data. The recorded images, gated over a given *m*/*z* peak, represent a two-dimensional projection of the three-dimensional scattering distribution. Abel inversion of these images using the pBASEX algorithm^44^ was used to reconstruct the underlying three-dimensional velocity distribution, from which kinetic energy distributions were derived. It is important to note that under velocity-mapping conditions, signal arising from particles with very low velocity (such as parent ions) is mapped to a very small region of the detector. This leads to overlapping clusters of bright pixels recorded by the PImMS camera, and thus individual ion hits can no longer be correctly identified by the hitfinding alogirithm. Furthermore, as pixels in the PImMS camera can only register four events per acquisition cycle, pixels at the center of the detector are susceptible to saturation by lighter fragments (which arrive prior to the I_2_^+^ ion) with low velocity, such as H_2_O^+^ background. In Figure 8a), these effects reduce the signal in the I_2_^+^ ion. To obtain more quantitative mass spectra, data was also recorded with spatially defocusing ion optics voltages, as presented in Figure S3 in the Supporting Information.

In order to determine the peak laser intensity at a given pulse energy, and also calibrate the VMI images, argon gas was ionized at a range of laser pulse energies. At each pulse energy, photoelectron spectra were recorded by operating the spectrometer in negative potential mode. From the observed ponderomotive shifts of the ATI peaks observed as a function of pulse energy, a reliable calibration to peak laser intensity could be made. The spacing of the ATI peaks observed (=1.55 eV, the photon energy) was also used to calibrate the energy scale of the velocity-map images.

## Results

### Photoion Imaging

**Figure 1:**
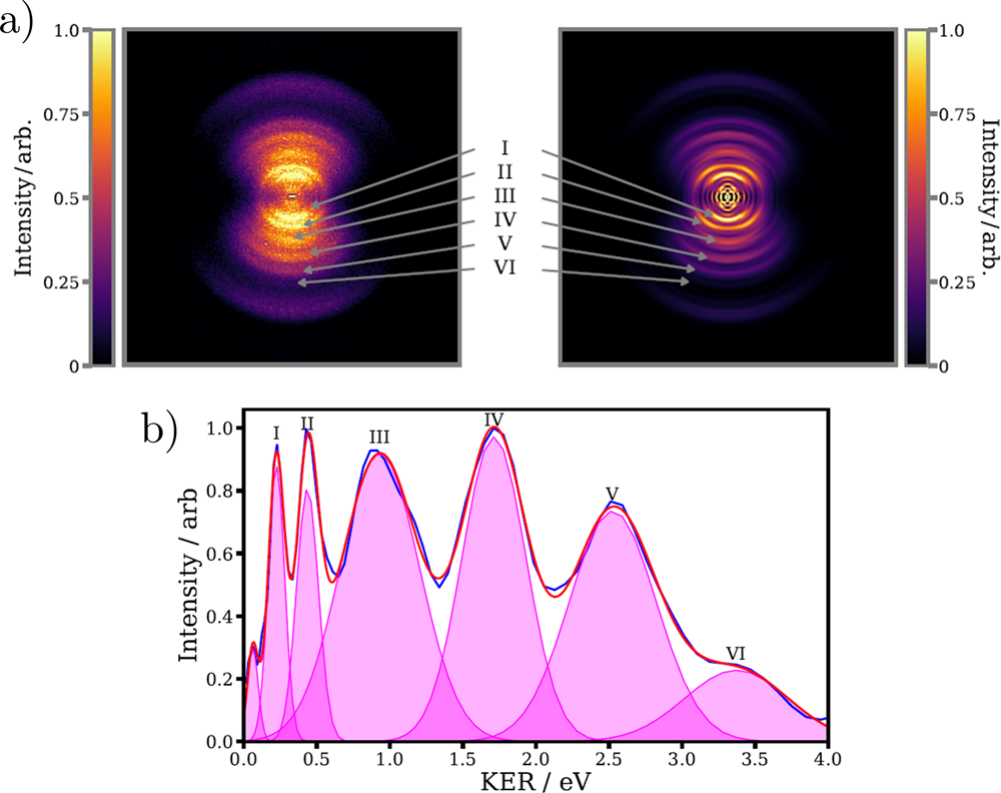
(a) Projected (left) and reconstructed central slice (right) velocity-map images for the I^+^ fragment produced following irradiation by ∼10^14^ Wcm^–2^ 800 nm pulses. The laser polarization axis is vertical in these images (and in all subsequent VMI images presented in this work). Six distinct channels produced by the dissociation of I_2_^+^ are observed and labeled I–VI. Features of higher kinetic energy originate from Coulomb explosion of higher I_2_ charge states. (b) Kinetic energy release distribution obtained from the reconstructed image shown in panel a. As discussed in the text, this distribution can be fit to multiple Gaussian contributions. Each Gaussian is shaded in magenta, given an overall fit distribution (red) which well reproduces the experimental distribution (blue).

An example velocity-map image for the I^+^ photoion is shown in Figure 1a), while the corresponding total Kinetic Energy Release (KER) spectrum is displayed in Figure 1b) (blue line). At least six distinct features arising predominantly from dissociation of I_2_^+^ into I^+^+I are observed, and labeled I–VI in order of increasing radius (i.e., I^+^ velocity). Signals at higher radius than feature VI originate from Coulomb explosion of multiply charged parent ions. This is confirmed conclusively through ion–ion covariance analysis^45,46^ (shown in the Supporting Information), and these Coulomb explosion channels will be discussed later in the manuscript. A very sharp feature at zero radius is also seen in the recorded image, which arises from undissociated I_2_^2+^ ions, which have identical mass-to-charge ratio as the I^+^ ions, but are produced with essentially zero KER, as observed previously.^34^ Immediately, the observation of a multitude of distinct photofragmentation pathways is surprising. In a previous velocity-mapping study of this system, some structure in the velocity distributions was observed, but the series of features could not be fully resolved.^34^ As discussed in more detail in the Supporting Information, we believe this is due to superior velocity resolution achieved in the current work.

If considering the photoionization process as a vertical excitation in the Franck–Condon region, the existence of these different channels implies that several dissociative electronic states of I_2_^+^ are produced following ionization and/or different product channels (i.e., electronic states of the I^+^ or I photofragments) are accessed.^47^ Further insights into the photoexcitation and fragmentation dynamics can be gained by examining the KER distribution of I^+^ ions, shown in Figure 1b). The overall spectrum may be fit to a series of Gaussian contributions representing the dissociation pathways (with an additional peak at almost zero KER to account for the aformentioned I_2_^2+^ contribution). The parameters for these Gaussian fits (centers, amplitudes and standard deviations) are given in Table 1. Removal of the HOMO or HOMO–1 electrons yield I_2_^+^ in the bound ^2^Π_*g*,3/2,1/2_, ^2^Π_*u*,3/2,1/2_ states.^48^ Ionizing the HOMO–2 to give I_2_^+^ in the ^2^Σ_*g*_^+^ state does lead to dissociation, but only to very low KER fragments (0–0.15 eV).^34^ The states which can lead to the observed higher KER channels therefore involve ionization of rather strongly bound orbitals of I_2_.

**Table 1:** Parameters from the Fit of the I^+^ KER Distribution, as Shown in Figure 1b)^a^

channel	center/eV	width (σ)/eV	amplitude
I	0.23 ± 0.01	0.05 ± 0.01	0.83 ± 0.11
II	0.43 ± 0.01	0.08 ± 0.01	0.80 ± 0.07
III	0.93 ± 0.02	0.26 ± 0.03	0.92 ± 0.04
IV	1.71 ± 0.02	0.22 ± 0.03	0.97 ± 0.05
V	2.53 ± 0.05	0.30 ± 0.07	0.74 ± 0.05
VI	3.37 ± 0.18	0.31 ± 0.21	0.23 ± 0.05

a Errors shown are statistical (1σ) fitting errors.

Precisely assigning the origins for these different photodissociation channels is challenging, as there are a multitude of dissociative electronic states which may be populated (and which are not fully characterized in the literature)^49^ and various product channels to which these states could correlate. The vertical ionization energies to several cationic states have been measured previously using electron momentum spectroscopy^50^ and photoelectron spectroscopy.^51^ Thus, asymptotic KERs for dissociation pathways can be predicted from energy conservation considerations using these vertical ionization energies along with known possible product internal energies:
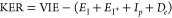
1where VIE is the relevant vertical ionization energy of I_2_, *E*_I_ and  are the internal energies of the I and I^+^ photoproducts respectively, *I*_*p*_ is the ionization potential of ground-state I atoms (10.45 eV^52^), and D_*e*_ is the dissociation energy of I_2_ (1.54 eV^53^). This description neglects the possibility of neutral dissociation prompted by (multiple) 800 nm photon absorption, followed by ionization of the resulting neutral iodine fragments. Given the short pulse duration employed in the current study, and the expected difficulty in populating low-lying dissociative states of I_2_ at 800 nm, we do not think such a process is a likely pathway. We note that this may not be the case at other wavelengths, as suggested by a previous study using intense 100 fs 610 nm fields, where neutral dissociation could be initiated on the neutral B state.^54^

**Figure 2:**
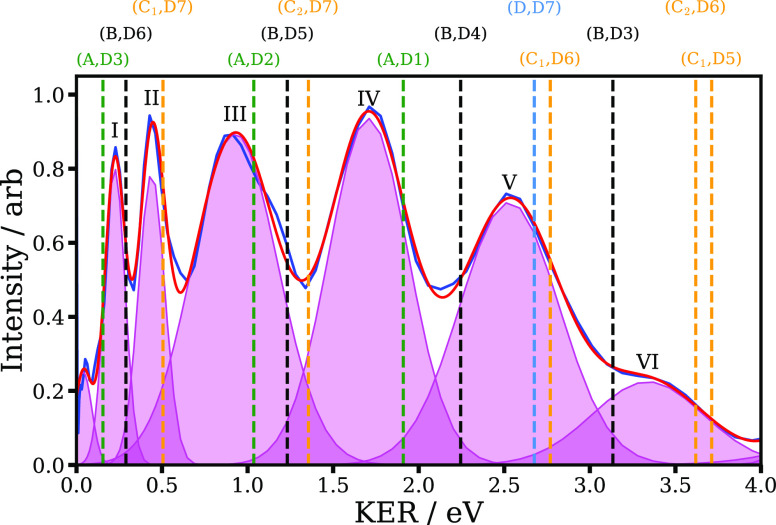
Comparison between the experimental (blue) and fitted (red) I^+^ KER distributions with predicted KERs for a range of possible fragmentation pathways, for different combinations of I_2_^+^ states (A, B, C_1_, C_2_, D) and product electronic states (D1, D2, D3, etc.). These predictions are discussed in more detail in the main text.

Figure 2 compares the experimental KER distribution with KERs predicted using Equation 1. The predicted KERs are labeled according to the electronic state of the I_2_^+^ (using the labeling of refs (49 and 50)) and the states of I and I^+^ products. A more detailed description of the KER prediction is given in the Supporting Information, including the electronic state of the I and I^+^ photoproducts associated with each dissociation limit (D1, D2, D3, etc.). It should be stressed that further work is required to identify the precise origins of the different fragmentation pathways observed in the current work. In particular, a more comprehensive theoretical characterization of the many low-lying electronic states of the I_2_^+^ cation would be invaluable.^49^ With this said, the comparisons shown in Figure 2 are informative. For instance, it can be seen that predicted dissociation channels from a single excited electronic state of I_2_^+^ are insufficient to explain the experimental observations, suggesting that multiple highly excited electronic states contribute to the strong-field fragmentation of I_2_^+^, supporting the claims made by previous work.^34^ The energetic widths of the different features (which can only be assessed with the higher-resolution measurements reported here) is also richly informative. Channels I and II exhibit significantly narrower KER distributions. This suggests that these dissociation pathways originate from cation potentials that are relatively shallow in the Franck–Condon region. Future work could aim to directly characterize the electronic states of the neutral I atom dissociation products for instance by photoelectron spectroscopy using a vacuum ultraviolet (VUV) pulse, to enable the definite assignment of dissociation pathways.

The observation of multiple dissociation channels could potentially have a number of origins in addition to direct ionization of deep-lying MOs. Excitation may occur following ionization, either due to electron rescattering^55^ or postionization excitation directly in the field.^30,56^ Previous work using circularly polarized light yielded no detectable changes in the measured velocity distributions, implying electron rescattering is unlikely to play an important role.^34^ The potential role of postionization excitation by the field cannot be conclusively ruled out in the current ion imaging data, but it will be explored more shortly in the context of photoelectron imaging results.

**Figure 3:**
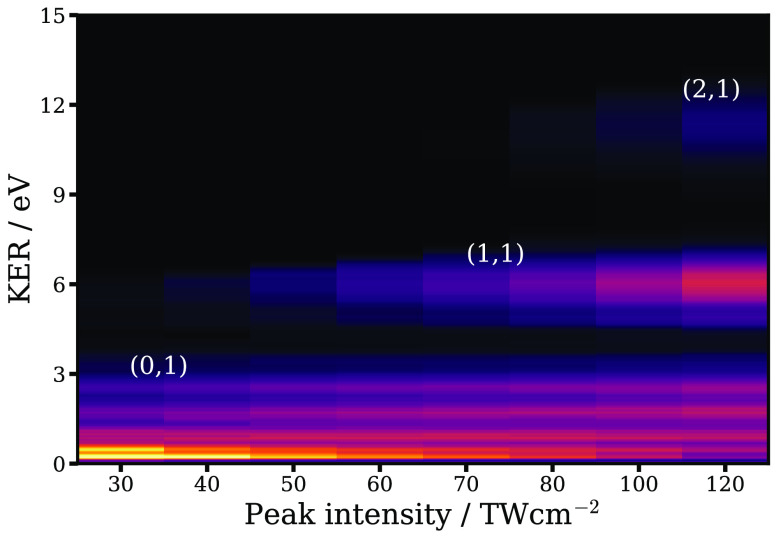
KER distributions (normalized to unit total intensity) extracted from a series of I^+^ VMI images as a function of peak laser intensity. Features assigned to fragmentation to (I + I^+^), (I^+^ + I^+^), and (I^2+^ + I^+^) are labeled (0,1), (1,1), and (2,1) respectively.

In order to examine the role of potential nuclear motion within the field (on field-distorted potentials), I^+^ KER distributions were recorded at a range of peak laser intensities (30–120 TW cm^–2^, corresponding to Keldysh parameters^57^ in the range 1.6 to 0.8). At different laser intensities, the extent of laser-induced couplings of potentials varies, as (likely) would the time spent on the potentials of the molecular cation during the field, with ionization becoming likely earlier in the pulse at higher peak intensities. We would expect these effects to lead to variations in the positions and widths of the individual KER spectra.^16,58^ Recorded I^+^ KERs as a function of peak laser intensity are shown in Figure 3. No such intensity-dependence in the dissociation ionization region is observed (as was the case in the previous lower-resolution imaging study on I_2_^34^), suggesting that nuclear motion of the heavy iodine atoms during the ionizing laser pulse does not play a large role in the observed asymptotic dissociation KERs. The main change with increasing peak laser intensity seen in Figure 3 is the increased prevalence of features with significantly higher KER, which originate from Coulomb explosion of multiply charged parent ions.

**Figure 4:**
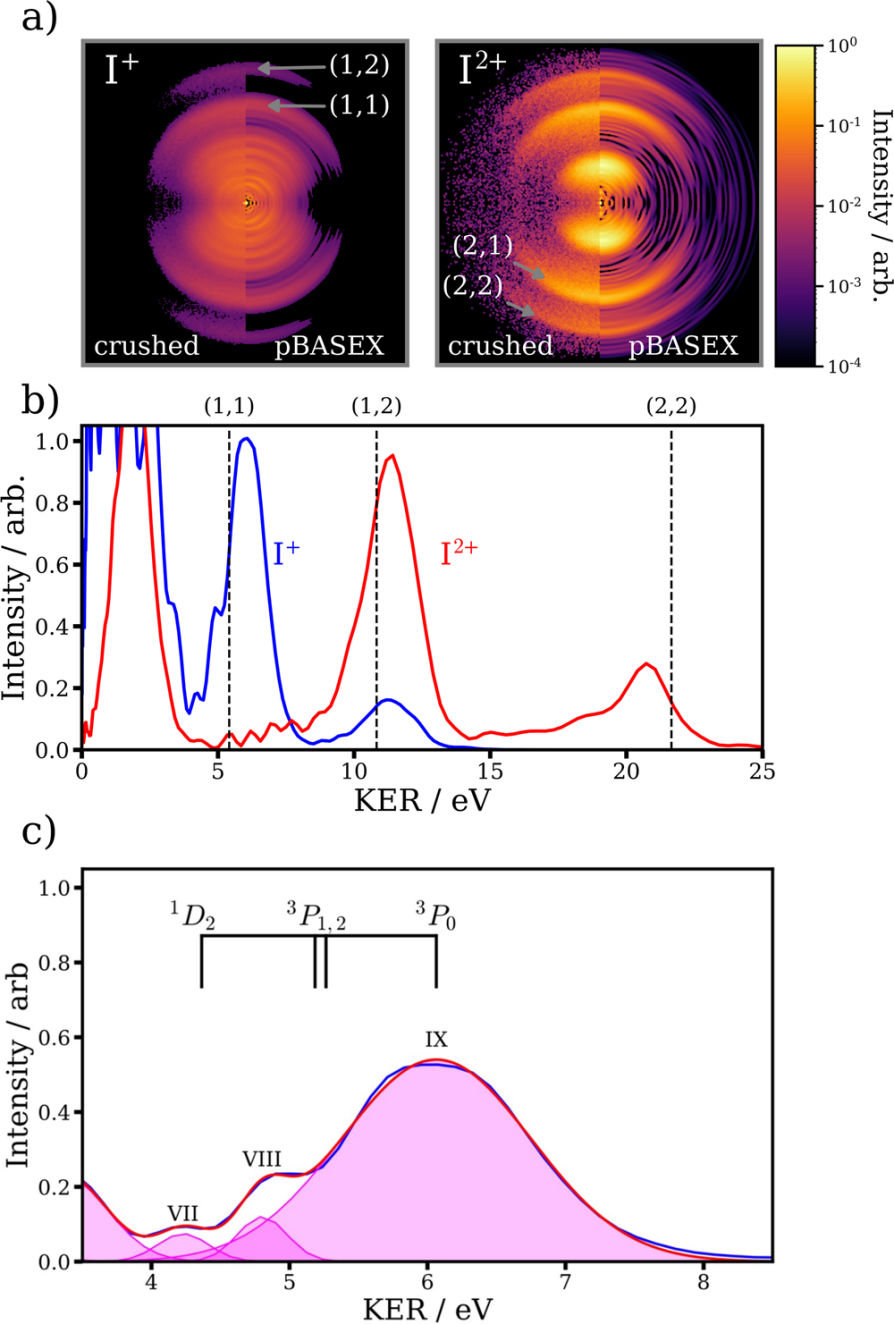
(a) VMI images for the I^+^ and I^2+^ ion. In both cases, the left-hand side is the recorded “crushed” image and the right-hand side is a slice image obtained following Abel inversion. A logarithmic color scale is used to enhance the visibility of the high radius features. (b) KER distributions extracted from the I^+^ (blue) and I^2+^ (red) ions. Dashed lines mark the predicted KERs for the Coulomb explosion channels assuming Coulombic repulsion at the equilibrium internuclear I–I bond distance. (c) Zoomed-in view of the KER distribution of the (1,1) Coulomb explosion channel (blue). A fit to the experimental data of a series of Gaussian contributions (plotted in magenta) is shown, which sum to give the fit distribution plotted in blue. Energetic splittings of the I^+^ product^52^ are also shown, as discussed in the main text.

Figure 4a displays sample I^+^ and I^2+^ VMI images with suitable logarithmic color scales to display the high KE features originating from Coulomb explosion. These features are labeled (*p*, *q*) according to the charge states of the two iodine ions (I^*p*+^, I^*q*+^) produced in the Coulomb explosion from a I_2_^(*p*+*q*)+^ polycation. These assignments are conclusively supported by covariance imaging analysis, which determines the correlated velocity distribution of a given pair of ions.^46,59^ Covariance imaging results are shown in the Supporting Information. In the I^2+^ image, significant contributions at much smaller radius than the (2,1) Coulomb explosion channel are seen. This signal arises from the charge asymmetric dissociation of I_2_^2+^ as has been studied in detail previously.^60,61^

Figure 4b displays the KER distributions extracted from the two images shown in panel a. Also marked are predicted KERs for the different Coulomb explosion pathways assuming purely Coulombic repulsion between two point charges at the ground state internuclear distance of I_2_. Reasonable agreement is seen for the different Coulomb explosion channels, although the (2,2) channel exhibits smaller KERs than predicted. Such behavior is commonly assigned to elongation of the bond during the ionization and fragmentation process,^19^ and/or to deviations of the true potentials of the molecular polycations from Coulombic behavior.^18^ Interestingly, the (1,1) Coulomb explosion feature in the I^+^ KER distribution appears to show some structure, with two shoulders at lower KER than the main feature. The (1,1) KER distribution is shown in an expanded view in Figure 4c), which can be well fit by three Gaussian functions. A similar splitting of the kinetic energy distribution has been observed in the (1,1) Coulomb explosion of methyl iodide.^62−64^ Supported by calculations of the *ab initio* potential energy curves of CH_3_I^2+^, this splitting could be assigned to contributions from different electronic states, which converge on different electronic states of the I^+^ product.^62^ While such calculations have not been performed in the current work, the energy splittings of low-lying states of the I^+^ cation are shown in Figure 4. The observed splittings are consistent with those exhibited between the I^+^^3^P_0_ ground state and the ^3^*P*_1,2_ and ^1^D_2_ excited states,^52^ but knowledge of the corresponding I_2_^2+^ potential energy curves in the Franck–Condon region would be required to conclusively make these assignments. It does appear that, much like in the dissociation of the monocation, Coulomb explosion of I_2_ involves contributions from a range of electronic states populated in the intense laser field.

**Figure 5:**
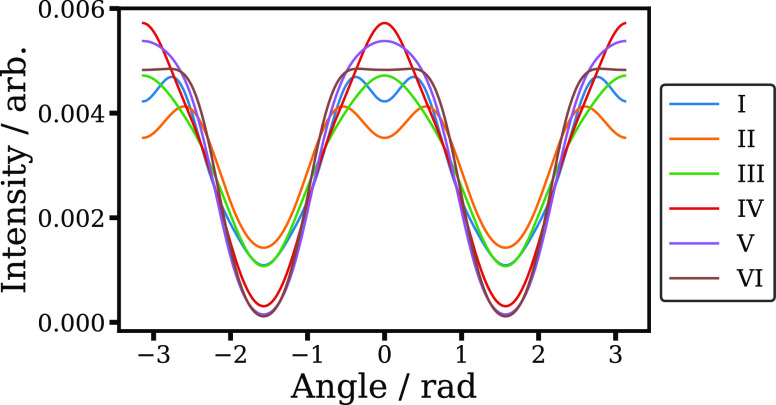
Photoion angular distributions for the different dissociative ionization channels identified in Figure 1. Here, intensity integrated over the radius of each feature is given as a function of angle relative to the laser polarization axis.

Finally, we consider the angular distributions associated with the VMI image shown in Figure 1a, which are plotted in Figure 5. The angular distributions associated with the I–VI channels are shown in Figure 5. The intensity distributions for all the dissociation channels peak along the laser polarization axis, as is typically observed in strong-field ionization fragmentation dissociation, due to geometric and dynamic alignment effects.^21,23^ There are, however, differences in the angular distributions of the different photofragmentation channels. In particular, channels I and II exhibit a “four-lobed” angular distribution, with local maxima either side of the laser polarization axis. Similar behavior was observed in a previous VMI study by Vrakking and co-workers^23^ (although individual channels could not be resolved as in the current work), and assigned to further ionization of molecules most strongly aligned to the laser polarization axis, causing a slight depletion in the yield of these ions very close to the laser polarization axis. This explanation is consistent with the observation that these channels become relatively less prominent at higher peak laser intensities (as seen in Figure 3). Other possible origins for the observed four-lobed angular distributions may include the combination of parallel and perpendicular transitions^65^ or tunneling from Π orbitals.^66^

### Photoelectron Imaging

**Figure 6:**
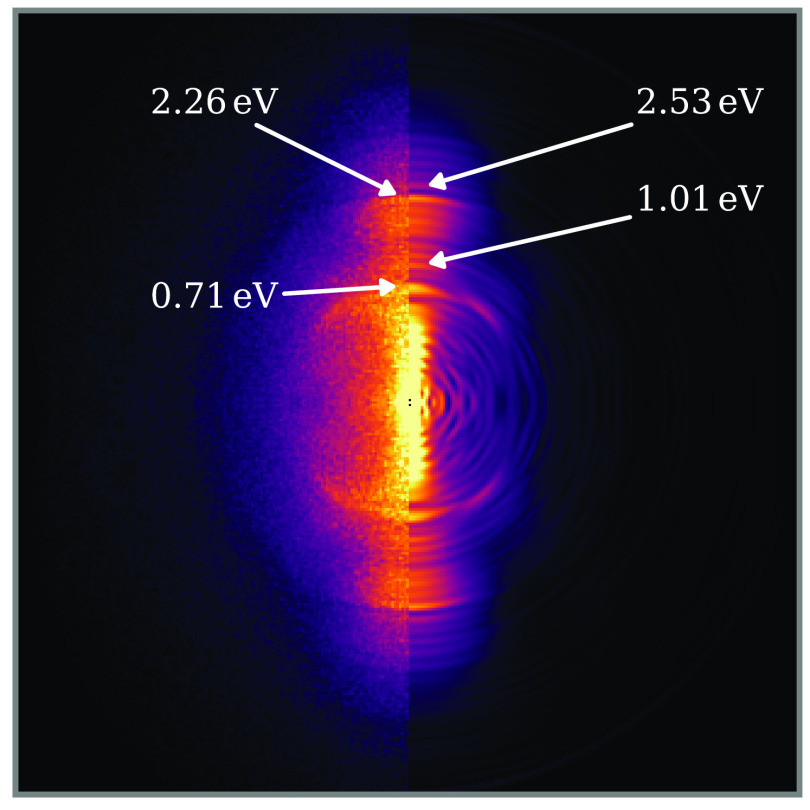
Example photoelectron image (left, recorded; right, reconstructed central slice) recorded following interaction of I_2_ molecules with NIR pulses with a peak intensity of 35 TW cm^–2^. The NIR laser is polarized along the vertical in this image. Several sharp features are labeled according to their electron kinetic energy. These features are discussed in detail in the main text.

In order to shed more light on the photoionization dynamics which underpin the photofragmentation dynamics of I_2_^+^ in strong fields, we turn to photoelectron spectroscopy. Figure 6 shows an example photoelectron image recorded following irradiation of I_2_ with 35 TW cm^–2^ NIR pulses, in which single ionization was the dominant process. Contributions from ionization of background gases (O_2_, N_2_, H_2_O) have been subtracted. In addition to indistinct signal spread over a broad range of energies, a number of sharp features are observed, several of which are labeled by their electron kinetic energy. The two most dominant features are observed at 0.71 and 1.01 eV, with further features occurring at approximately 1.55 eV (the photon energy) higher electron kinetic energy (eKE), arising from the next ATI order. Nonresonant ionization features are blurred by differing ponderomotive shifts of the ionic continua within the intensity profile of the focused pulse. Features with well-defined eKE are indicative of Freeman resonances—ionization via a resonant excitation state to a Stark-shifted Rydberg state.^35^ Since their first observation in rare gas atoms, Freeman resonances have since been identified in a series of species, including several small molecules.^24,67−69^

**Figure 7:**
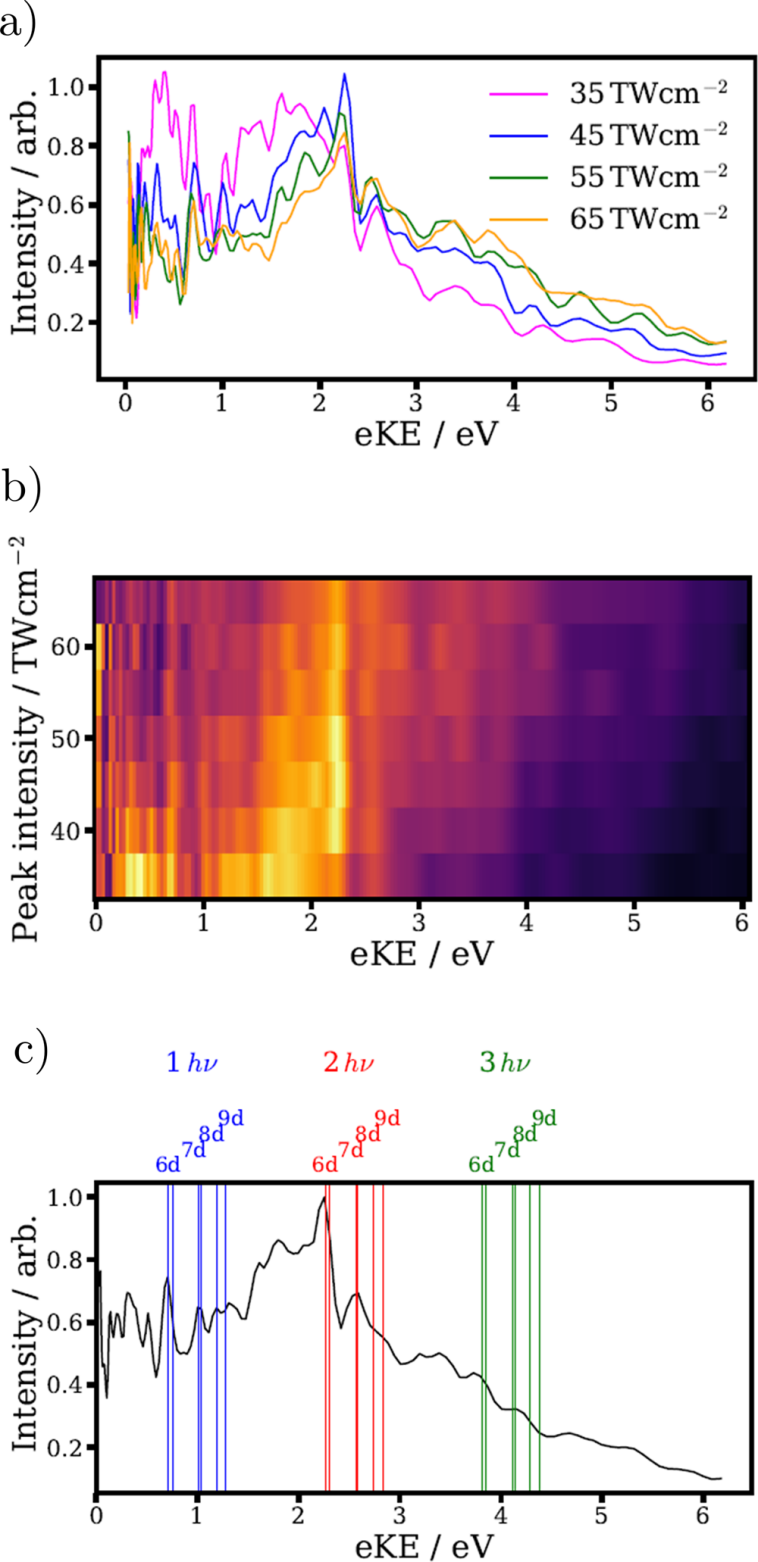
(a) Recorded photoelectron spectra at a series of peak laser intensities, as labeled. (b) Alternative two-dimensional representation of the intensity-dependent photoelectron spectra. (c) Photoelectron spectra averaged over the different intensities shown in panel b. The expected kinetic energies for ionization (to the ground state of the cation) via resonant excitation to different *n*d Rydberg states are shown, assuming one (blue), two (red), or three (green) photons are involved in the ionization step.

In order to further examine the role of resonant and nonresonant ionization pathways, photoelectron images were recorded over a range of peak laser intensities, results of which are shown in Figure 7. At the intensity range studied, single ionization of I_2_ was dominant, mainly producing stable I_2_^+^ or low velocity I^+^ as observed in ion mass spectra. Except for the sharp features originating from Freeman resonances, the photoelectron kinetic energy spectra are generally rather structureless, with no clear peaks from individual nonresonant ionization pathways. Ionization to a single continuum would give rise to a series of broad ATI peaks. The absence of such structure can be understood if there are several prominent nonresonant ionization pathways to different ionic continua. The general structure of the recorded photoelectron spectra suggests that direct ionization of deep-lying MOs is a major contributor to the observed cation photofragmentation pathways, as opposed to initial ionization to lower-lying states of the cation which are then further excited in the field.

While the majority of the photoelectron signal in Figure 7 can be assigned to nonresonant ionization to multiple ionic continua, several sharp features are present on top of this broad signal. Such features, whose positions do not vary, are indicative of Freeman resonances, as mentioned previously. Under the assumption that the neutral Rydberg state and the final cationic state experience the same ponderomotive shifts, electron kinetic energies are determined by the field-free (rovibonic) energies of the intermediate Rydberg state *E*_Rydberg_, the ion state *E*_ion_, and the number of photons absorbed in the ionization step, *n*:^35,69^
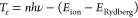
2

In principle, differences in vibrational energy between the ion and intermediate Rydberg state would lead to a broadening of the photoelectron spectrum. However, there is a very strong propensity for ionizations where Δ*v* = 0 due to near-perfect Franck–Condon overlap, and the vibrational constants of ionic and Rydberg states are very similar. As such, Freeman resonances lead to sharp photoelectron kinetic energy distributions, even in molecules.^24^

Figure 7c compares the laser intensity-averaged photoelectron spectrum and predicted electron kinetic energies using eq 2 for literature energies of high-lying Rydberg states with even *l* converging on the ^2^Π_*g*,3/2_ ground state of I_2_^+^, measured by (2 + 1) Resonance-Enhanced Multiple Photon Ionization Spectroscopy (REMPI).^70,71^ While resonant contributions to ionization to other excited states of the cation are likely to occur, we focus solely on the ionic ground state, which we believe is responsible for the most common resonant ionization pathways at these low peak laser intensities. Contributions from odd *l* Rydberg states can be neglected, as the  ground state of I_2_ can only couple to *g* (*u*) parity excited states when absorbing an even (odd) number of photons.^67^ Odd *l* Rydberg states would therefore need to experience a greater ponderomotive shift to bring them into a 7-photon resonance, relative to the shift required to bring the even *l* states into a 6-photon resonance to contribute to any resonant ionization processes. For Rydberg states which are consistent with the observed photoelectron energies, intensities of ∼30–40 TW cm^–2^ are required to bring them into 7-photon resonance. The fact that all the prominent resonant features are still observed strongly at 35 TW cm^–2^, suggest that contributions from odd *l* Rydberg states are not significant. To confirm this conclusively, extension of the present study to lower peak intensities would be required.

Good agreement is seen between the predictions and the observed peak positions in the experimental spectrum, especially for the two most prominent Freeman resonances, matching predictions for ionization via the 6d and 7d molecular Rydberg states. Figure 7 also shows that the main Freeman resonances are observed at all the peak intensities studied. This is consistent with calculating the ponderomotive shift (and therefore minimum laser intensity) required to shift these Rydberg levels into 6-photon resonance with the 800 nm field. These intensities are given in Table 2, and are all lower than the lowest peak intensity used in the current study. To further disentangle the resonant ionization pathways, it would be interesting to examine lower peak intensities (which was challenging in the present study due to low signal rates), to access intensity regions at which certain Freeman resonances cannot occur. This would strengthen the current assignment, and could potentially allow the identification of other resonant features in the photoelectron spectrum. For instance, in the spectra shown in Figure 7 there are indications of sharp peaks in the 0–0.7 eV which are not assigned in the current work.

**Table 2:** Literature Energies of I_2_ Rydberg States Believed to Be Involved in the Freeman Resonances Observed in the Experimental Photoelectron Spectra^a^

Rydberg State	energy/eV	resonant intensity/TW cm^–2^
6d	8.47	13.9
7d	8.77	8.8
8d	8.95	5.9
9d	9.04	4.3

a The predicted photoelectron energies for ionization via these states are shown in Figure 7c). For each Rydberg state, the intensity required to ponderomotively shift the transition into 6-photon resonance with the 800 nm laser field is also given. The energies of the Rydberg states are taken from ref. (70). Only the Rydberg states with ω (total angular momentum projection quantum number) of 2 are shown as the variation of Rydberg state energy with ω is relatively small. Note that we are using the more recent reassignment of these Rydberg features given in ref (71). Previously (in ref (70)), the features now assigned to *n*d Rydberg states were incorrectly assigned to (*n* + 2)s Rydberg states.

We would like to stress that the ^2^Π molecular cations formed by resonant ionization will remain bound, in the absence of any postionization excitation. While resonant ionization gives very clear signatures in the photoelectron spectra, the majority of ionization occurs nonresonantly, and is believed to be responsible for the dissociative cationic states. A relatively small, but still significant portion of ionization to bound cationic states is consistent with the yield of I_2_^+^ in the ion mass spectra (shown in Figure S3 of the Supporting Information).

A promising avenue for future work would be to image photoelectrons and photoions in coincidence (or covariance^30,45,72^), and to study the photoelectron spectra correlated to individual dissociation channels. As demonstrated in the recent work of Weinacht and co-workers studying strong-field ionized D_2_O, channel-resolved ATI spectra would show characteristic shifts for the different electronic states involved, if these states are populated directly following ionization.^31^ Employing such a scheme to determine the photoelectron spectrum correlated to the individual photofragmentation channels of I_2_^+^ observed in the present work could therefore be particularly illuminating. Such an experiment would determine the important relationship between which MO is ionized and the ultimate photofragmentation pathway.

### Photoanion Imaging

The photoelectron data presented in the current work establishes the important role of intermediate Rydberg states of the neutral during the strong-field ionization of I_2_. This raises the question of whether any neutral Rydberg states remain populated after the pulse. Previously it has been established that, following interaction with an intense laser field, population may remain “trapped” in high-lying neutral Rydberg states.^73,74^ A common explanation for this phenomenon is the high centrifugal barrier such Rydberg states may possess due to their high angular momentum. In the case of molecules, these Rydberg states may have important consequences for the ultimate photofragmentation dynamics, for instance in producing neutral atomic Rydberg fragments following dissociation of these molecular Rydberg states (as observed in H_2_^75^ and O_2_^76,77^). In the case of O_2_, recent work has identified dissociation channels of molecular Rydberg states specifically populated by resonant multiphoton excitation.^76^

To identify possible consequences of resonantly excited neutral molecular Rydberg state population in I_2_, we turn to the extensive literature on the properties of I_2_ Rydberg states. It has been well established that many such Rydberg states act as “doorways” to ion-pair states,^37,39,48,70,78,79^ which, assuming the dissociation of the ion-pair state is exceeded, can produce I^+^ + I^–^ photofragment pairs. Baklanov and co-workers have recently conducted nanosecond^36^ and femtosecond^38^ velocity-map imaging studies on the photofragmentation dynamics of 6d Rydberg states of I_2_. These experiments identified a number of dissociation channels mediated by ion-pair states, either directly to ion pair products or to electronically excited atoms through lower-lying Rydberg states. As the photoelectron spectra shown in Figure 7c exhibit prominent features assigned to ionization via 6d (and higher) Rydberg states, like those studied by Baklanov and co-workers, we conducted a search for anionic products from ion pair dissociations.

**Figure 8:**
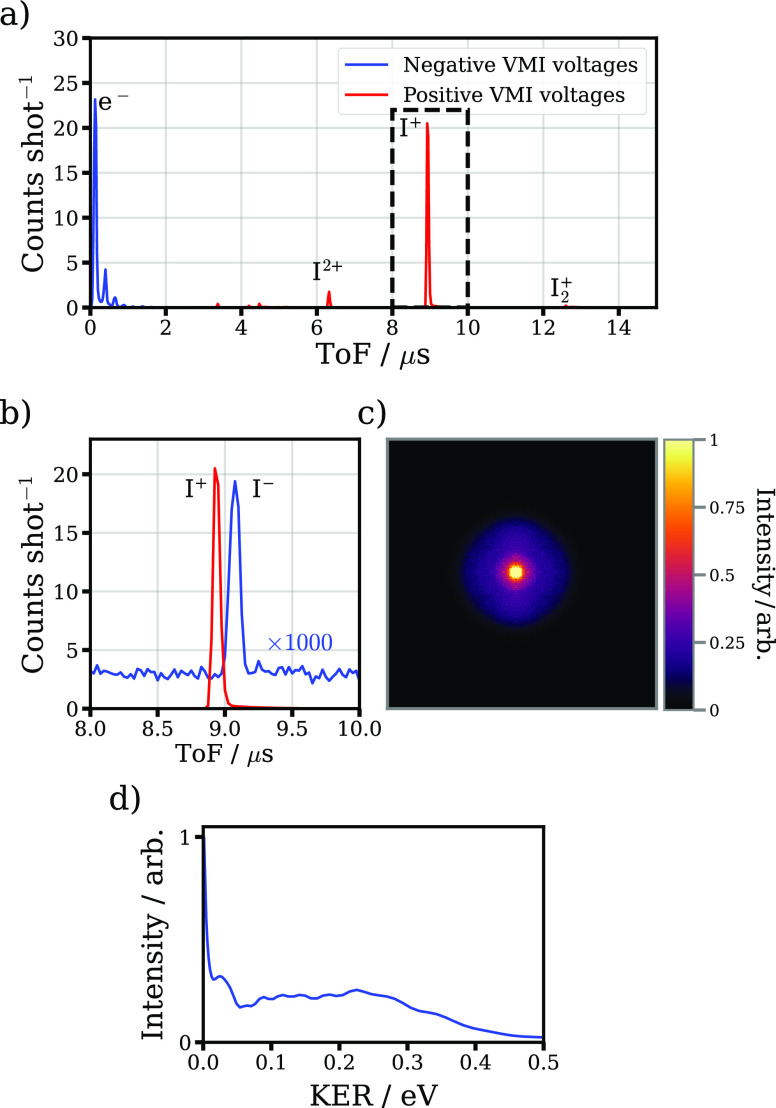
Summary of the photoanion imaging data. (a) Time-of-flight spectra for I_2_ upon irradiation by the 800 nm laser at a peak intensity of 60 TW cm^–2^, with the spectrometer operating in positive (red) and negative (blue) voltage mode. (b) Expanded view at the time-of-flight region corresponding the I^+^ ion. By multiplying the negative voltage signal by a factor of 1000, a clear peak corresponding to the I^–^ ion is seen. (c) VMI image of the I^–^ ion. (d) KER distribution for the I^–^ ion extracted by Abel inversion of the VMI image.

When operating the VMI spectrometer in negative potential mode in order to image photoelectrons, photoanions are also accelerated under velocity-mapping conditions. The use of a fast timestamping camera allows the simultaneous imaging of photoelectrons and photoanions, with the latter recorded at significantly later times-of-flight than the prompt electron signal. We observe a (very weak) signal from anions, specifically the I^–^ ion, which we believe originates from ion-pair dissociations from neutral Rydberg states. Figure 8a) compares the time-of-flight spectrum recorded for I_2_ under both spectrometer conditions. If the negative voltage spectrum is appropriately magnified and zoomed, as shown in panel b, a clear peak is seen at approximately the same time-of-flight that I^+^ ions are observed in the positive voltage case, suggesting production of I^–^ ions. A slight discrepancy between the two peaks is observed, which we believe is due to slight miscalibration of the power supplies used to switch the ion optics between positive and negative potentials. It should be noted that there is no analogous signal at the expected time-of-flight of any other possible anions (e.g., I_2_^–^). The very small relative magnitude of the I^–^ signal implies that the process leading to anion production is far less likely than fragmentations producing I^+^ cations. Given the low energetic threshold for I^–^ photodetatchment (3.06 eV,^80^ about two 800 nm photons), it may be possible for I^–^ anions produced in the field to be subsequently photodetatched, weakening the I^–^ signal observed.^36^ As the peak laser intensity is increased, the intensity of the I^–^ feature decreases relative to the total electron yield (see Figure S6 in the Supporting Information), which may be due to an increased probability for photodetatchment, or an increased probability for ionization of the Rydberg state.

Panel c shows the image associated with the I^–^ ion. For this data, the absolute voltage of the repeller was reduced to magnify the image associated with the I^–^ ions, which are produced with low velocity. In contrast to the cation and electron images produced, the I^–^ image is essentially isotropic. There are also indications of channels with distinct kinetic energies, as highlighted in panel d, which displays the KER distribution extracted from Abel inverting the image shown in panel c. A very sharp feature with zero KER is observed, along with a peak at ∼0.03 eV KER, and a far broader peak centered at ∼0.25 eV KER.

Previous work studying anion production from gas-phase molecules in intense laser fields is scarce. Molecular and fragment anion formed by irradiating small molecules (CO_2_, CS_2_, O_2_) in intense laser fields has been reported in one study, an observation assigned to attachment by low energy electrons produced in the field.^81^ In this previous study, both parent and fragment anions were observed, believed to come from nondissociative and dissociative electron attachment, respectively. The fact that, in the current work, only I^–^ ions are seen implies a different origin, as does the fact that, under the conditions of our experiment, no anions are produced from any background gases (O_2_, N_2_, H_2_O), or from the Ar sample used to calibrate the spectrometer.

The isotropic I^–^ velocity distribution seen in Figure 8c would be expected if the Rydberg states are populated via intermediate states of different symmetries,^36^ or if ion-pair dissociation is rather slow relative to the time scale of molecular rotation (albeit fast relative to the microsecond time scale of the time-of-flight measurement). This may be due to the time taken for population transfer from the initially prepared (bound) Rydberg states to the relevant dissociative ion-pair states. Recent work studying the dissociation dynamics of I_2_ 6d Rydberg states populated by two-photon absorption in the ultraviolet have identified several such predissociation pathways with picosecond lifetimes.^36,38^ This study reported I^–^/I^+^ ion-pair dissociation products with ∼0.25 eV KER and an approximately isotropic angular distribution, in qualitative agreement with the higher KER feature observed in Figure 8, parts c and d. The significant broadness of this feature in the current data, in addition to the additional channels at much lower KER suggest the existence of multiple ion-pair dissociations, consistent with the observation of several Rydberg states of higher energy than the 6d the photoelectron spectra (Figure 7c).

Future work more thoroughly exploring this proposed pathway for anion production in intense laser fields would be of great interest, for instance by extending the current study to other small halogen-containing molecules or to study anion formation and subsequent photodetatchment in a time-resolved manner. A thorough study of the dependence of the ion-pair dissociation KER spectra on laser intensity and pulse duration may also be illuminating, to asses how Stark shifts of the high-lying Rydberg states and the ion-pair states affect the dissociation dynamics. The single-sided velocity-map imaging spectrometer used here can only be used to measure either cations or anions in a given experimental cycle. In principle, future work using a dual-sided velocity-map imaging spectrometer could record anions and cations in coincidence. The coincident detection of I^–^ and I^+^ anions with equal and opposite momenta would conclusively determine their origin as ion-pair dissociations of neutral I_2_ molecules. In practice such measurements may be challenging however, given the vast overlapping background of I^+^ ions formed by cation fragmentation.

## Conclusions

In conclusion, we have presented an experimental study of the photoionization and photofragmentation of I_2_ molecules in intense femtosecond NIR laser fields, which reveal a range of interesting and complex dynamics. In particular, many different ionization pathways are believed to be in operation, leaving the I_2_^+^ cation in multiple electronic states, as evidenced by distinct features in the ion KER distributions. At higher peak laser fields, Coulomb explosion of multiply charged parent ions is observed. The structures of the associated kinetic energy distributions again imply the existence of multiple fragmentation pathways originating from different electronic states. Photoelectron measurements provide supporting evidence that direct nonresonant ionization from several deeply bound MOs is responsible for these fragmentation channels. Clear photoelectron signal assignable to resonant ionization to low-lying, bound cationic states was also observed, and several intermediate Rydberg states identified. Interestingly, I^–^ anions were detected, which originate from several ion-pair dissociation pathways of these Rydberg states which can remain populated in the field. This is the first observation of anion production in intense laser fields by this mechanism, but may be a more general phenomena in molecules with strongly coupled Rydberg and ion pair states.^79^

## Supporting Information Available

Detailed comparison of the I^+^ image to that reported in the literature, a summary of additional covariance imaging analysis, detailed description of the predicted KERs for I_2_^+^ dissociation channels, an example of an ion mass spectrum, and further analysis of the laser intensity-dependent cation and anion imaging data (PDF)

## References

[ref1] Stapelfeldt H.; Constant E.; Corkum P. B. Wave Packet Structure and Dynamics Measured by Coulomb Explosion. Phys. Rev. Lett. 1995, 74, 3780–3783. 10.1103/PhysRevLett.74.3780. 10058295

[ref2] Allum F.; Burt M.; Amini K.; Boll R.; Köckert H.; Olshin P. K.; Bari S.; Bomme C.; Brauße F.; Cunha de Miranda B.; et al. Coulomb explosion imaging of CH_3_I and CH_2_ClI photodissociation dynamics. J. Chem. Phys. 2018, 149, 204313 10.1063/1.5041381. 30501230

[ref3] Ding X.; Forbes R.; Kübel M.; Lee K. F.; Spanner M.; Naumov A. Y.; Villeneuve D. M.; Stolow A.; Corkum P. B.; Staudte A. Threshold photodissociation dynamics of NO_2_ studied by time-resolved cold target recoil ion momentum spectroscopy. J. Chem. Phys. 2019, 151, 174301 10.1063/1.5095430. 31703482

[ref4] Endo T.; Neville S. P.; Wanie V.; Beaulieu S.; Qu C.; Deschamps J.; Lassonde P.; Schmidt B. E.; Fujise H.; Fushitani M.; et al. Capturing roaming molecular fragments in real time. Science 2020, 370, 1072–1077. 10.1126/science.abc2960. 33243885

[ref5] Itatani J.; Levesque J.; Zeidler D.; Niikura H.; Pépin H.; Kieffer J. C.; Corkum P. B.; Villeneuve D. M. Tomographic imaging of molecular orbitals. Nature 2004, 432, 867–871. 10.1038/nature03183. 15602553

[ref6] Li W.; Zhou X.; Lock R.; Patchkovskii S.; Stolow A.; Kapteyn H. C.; Murnane M. M. Time-Resolved Dynamics in N2O4 Probed Using High Harmonic Generation. Science 2008, 322, 1207–1211. 10.1126/science.1163077. 18974317

[ref7] Smirnova O.; Mairesse Y.; Patchkovskii S.; Dudovich N.; Villeneuve D.; Corkum P.; Ivanov M. Y. High harmonic interferometry of multi-electron dynamics in molecules. Nature 2009, 460, 972–977. 10.1038/nature08253. 19626004

[ref8] Haessler S.; Caillat J.; Boutu W.; Giovanetti-Teixeira C.; Ruchon T.; Auguste T.; Diveki Z.; Breger P.; Maquet A.; Carré B.; et al. Attosecond imaging of molecular electronic wavepackets. Nat. Phys. 2010, 6, 200–206. 10.1038/nphys1511.

[ref9] Wörner H. J.; Bertrand J. B.; Corkum P. B.; Villeneuve D. M. High-harmonic homodyne detection of the ultrafast dissociation of Br 2 molecules. Phys. Rev. Lett. 2010, 105, 103002 10.1103/PhysRevLett.105.103002. 20867516

[ref10] Wörner H. J.; Bertrand J. B.; Fabre B.; Higuet J.; Ruf H.; Dubrouil A.; Patchkovskii S.; Spanner M.; Mairesse Y.; Blanchet V.; et al. Conical intersection dynamics in NO2 probed by homodyne high-harmonic spectroscopy. Science 2011, 334, 208–212. 10.1126/science.1208664. 21998383

[ref11] Zuo T.; Bandrauk A.; Corkum P. Laser-induced electron diffraction: a new tool for probing ultrafast molecular dynamics. Chem. Phys. Lett. 1996, 259, 313–320. 10.1016/0009-2614(96)00786-5.

[ref12] Blaga C. I.; Xu J.; Dichiara A. D.; Sistrunk E.; Zhang K.; Agostini P.; Miller T. A.; Dimauro L. F.; Lin C. D. Imaging ultrafast molecular dynamics with laser-induced electron diffraction. Nature 2012, 483, 194–197. 10.1038/nature10820. 22398558

[ref13] Wolter B.; Pullen M. G.; Le A.-T.; Baudisch M.; Doblhoff-Dier K.; Senftleben A.; Hemmer M.; Schröter C. D.; Ullrich J.; Pfeifer T.; et al. Ultrafast electron diffraction imaging of bond breaking in di-ionized acetylene. Science 2016, 354, 308–312. 10.1126/science.aah3429. 27846561

[ref14] Amini K.; Sclafani M.; Steinle T.; Le A. T.; Sanchez A.; Müller C.; Steinmetzer J.; Yue L.; Martínez Saavedra J. R.; Hemmer M.; et al. Imaging the Renner–Teller effect using laser-induced electron diffraction. Proc. Natl. Acad. Sci. U.S.A. 2019, 116, 8173–8177. 10.1073/pnas.1817465116. 30952783 PMC6486720

[ref15] Ibrahim H.; Lefebvre C.; Bandrauk A. D.; Staudte A.; Légaré F. H2: the benchmark molecule for ultrafast science and technologies. Journal of Physics B: Atomic, Molecular and Optical Physics 2018, 51, 042002 10.1088/1361-6455/aaa192.

[ref16] Bucksbaum P. H.; Zavriyev A.; Muller H. G.; Schumacher D. W. Softening of the H_2_^+^ molecular bond in intense laser fields. Phys. Rev. Lett. 1990, 64, 1883–1886. 10.1103/PhysRevLett.64.1883. 10041519

[ref17] Yao G.; Chu S. I. Laser-induced molecular stabilization and trapping and chemical bond hardening in intense laser fields. Chem. Phys. Lett. 1992, 197, 413–418. 10.1016/0009-2614(92)85793-A.

[ref18] Corrales M. E.; González-Vázquez J.; Balerdi G.; Solá I. R.; de Nalda R.; Bañares L. Control of ultrafast molecular photodissociation by laser-field-induced potentials. Nat. Chem. 2014, 6, 785–790. 10.1038/nchem.2006. 25143213

[ref19] Zuo T.; Bandrauk A. D. Charge-resonance-enhanced ionization of diatomic molecular ions by intense lasers. Phys. Rev. A 1995, 52, R2511 10.1103/PhysRevA.52.R2511. 9912637

[ref20] Seideman T.; Ivanov M. Y.; Corkum P. B. Role of electron localization in intense-field molecular ionization. Physical review letters 1995, 75, 2819 10.1103/PhysRevLett.75.2819. 10059413

[ref21] Posthumus J. H.; Plumridge J.; Thomas M. K.; Codling K.; Frasinski L. J.; Langley A. J.; Taday P. F. Dynamic and geometric laser-induced alignment of molecules in intense laser fields. Journal of Physics B: Atomic, Molecular and Optical Physics 1998, 31, L553 10.1088/0953-4075/31/13/002.

[ref22] Ellert C.; Corkum P. B. Disentangling molecular alignment and enhanced ionization in intense laser fields. Physical Review A - Atomic, Molecular, and Optical Physics 1999, 59, R3170–R3173. 10.1103/PhysRevA.59.R3170.

[ref23] Rosca-Pruna F.; Springate E.; Offerhaus H. L.; Krishnamurthy M.; Farid N.; Nicole C.; Vrakking M. J. J. Spatial alignment of diatomic molecules in intense laser fields: I. Experimental results. Journal of Physics B: Atomic, Molecular and Optical Physics 2001, 34, 4919 10.1088/0953-4075/34/23/332.

[ref24] Gibson G. N.; Freeman R. R.; McIlrath T. J. Dynamics of the high-intensity multiphoton ionization of N_2_. Phys. Rev. Lett. 1991, 67, 1230 10.1103/PhysRevLett.67.1230. 10044093

[ref25] Akagi H.; Otobe T.; Staudte A.; Shiner A.; Turner F.; Dörner R.; Villeneuve D. M.; Corkum P. B. Laser Tunnel Ionization from Multiple Orbitals in HCl. Science 2009, 325, 1364–1367. 10.1126/science.1175253. 19745145

[ref26] Liu H.; Zhao S.-F.; Li M.; Deng Y.; Wu C.; Zhou X.-X.; Gong Q.; Liu Y. Molecular-frame photoelectron angular distributions of strong-field tunneling from inner orbitals. Phys. Rev. A 2013, 88, 061401 10.1103/PhysRevA.88.061401.

[ref27] Sándor P.; Zhao A.; Rozgonyi T.; Weinacht T. Strong field molecular ionization to multiple ionic states: direct versus indirect pathways. Journal of Physics B: Atomic, Molecular and Optical Physics 2014, 47, 124021 10.1088/0953-4075/47/12/124021.

[ref28] Zhao A.; Sándor P.; Rozgonyi T.; Weinacht T. Removing electrons from more than one orbital: direct and indirect pathways to excited states of molecular cations. Journal of Physics B: Atomic, Molecular and Optical Physics 2014, 47, 204023 10.1088/0953-4075/47/20/204023.

[ref29] McFarland B. K.; Farrell J. P.; Bucksbaum P. H.; Gühr M. High Harmonic Generation from Multiple Orbitals in N_2_. Science 2008, 322, 1232–1235. 10.1126/science.1162780. 18974318

[ref30] Boguslavskiy A. E.; Mikosch J.; Gijsbertsen A.; Spanner M.; Patchkovskii S.; Gador N.; Vrakking M. J.; Stolow A. The multielectron ionization dynamics underlying attosecond strong-field spectroscopies. Science 2012, 335, 1336–1340. 10.1126/science.1212896. 22422980

[ref31] Cheng C.; Forbes R.; Howard A. J.; Spanner M.; Bucksbaum P. H.; Weinacht T. Momentum-resolved above-threshold ionization of deuterated water. Phys. Rev. A 2020, 102, 052813 10.1103/PhysRevA.102.052813.

[ref32] Chandler D. W.; Houston P. L. Two-dimensional imaging of state-selected photodissociation products detected by multiphoton ionization. J. Chem. Phys. 1987, 87, 1445–1447. 10.1063/1.453276.

[ref33] Eppink A. T. J. B.; Parker D. H. Velocity map imaging of ions and electrons using electrostatic lenses: Application in photoelectron and photofragment ion imaging of molecular oxygen. Rev. Sci. Instrum. 1997, 68, 3477–3484. 10.1063/1.1148310.

[ref34] Smith D. L.; Tagliamonti V.; Dragan J.; Gibson G. N. Single ionization of molecular iodine. Phys. Rev. A 2017, 95, 1–8. 10.1103/PhysRevA.95.013410.

[ref35] Freeman R. R.; Bucksbaum P. H.; Milchberg H.; Darack S.; Schumacher D.; Geusic M. E. Above-threshold ionization with subpicosecond laser pulses. Phys. Rev. Lett. 1987, 59, 1092–1095. 10.1103/PhysRevLett.59.1092. 10035138

[ref36] Bogomolov A. S.; Grüner B.; Kochubei S. A.; Mudrich M.; Baklanov A. V. Predissociation of high-lying Rydberg states of molecular iodine via ion-pair states. J. Chem. Phys. 2014, 140, 124311 10.1063/1.4869205. 24697445

[ref37] Donovan R. J.; Lawley K. P.; Ridley T. Heavy Rydberg behaviour in high vibrational levels of some ion-pair states of the halogens and inter-halogens. J. Chem. Phys. 2015, 142, 204306 10.1063/1.4921560. 26026446

[ref38] Von Vangerow J.; Bogomolov A. S.; Dozmorov N. V.; Schomas D.; Stienkemeier F.; Baklanov A. V.; Mudrich M. Role of ion-pair states in the predissociation dynamics of Rydberg states of molecular iodine. Phys. Chem. Chem. Phys. 2016, 18, 18896–18904. 10.1039/C6CP02160C. 27353150

[ref39] Mulliken R. S. The halogen molecules and their spectra. J-J-like coupling. Molecular ionization potentials. Phys. Rev. 1934, 46, 549–571. 10.1103/PhysRev.46.549.

[ref40] Amini K.; Blake S.; Brouard M.; Burt M. B.; Halford E.; Lauer A.; Slater C. S.; Lee J. W.; Vallance C. Three-dimensional imaging of carbonyl sulfide and ethyl iodide photodissociation using the pixel imaging mass spectrometry camera. Rev. Sci. Instrum. 2015, 86, 103113 10.1063/1.4934544. 26520946

[ref41] Allum F.; Mason R.; Burt M.; Slater C. S.; Squires E.; Winter B.; Brouard M. Post extraction inversion slice imaging for 3D velocity map imaging experiments. Mol. Phys. 2021, 119, e1842531 10.1080/00268976.2020.1842531.

[ref42] Nomerotski A.; Adigun-Boaye S.; Brouard M.; Campbell E.; Clark A.; Crooks J.; John J. J.; Johnsen A. J.; Slater C.; Turchetta R.; et al. Pixel imaging mass spectrometry with fast silicon detectors. Nuclear Instruments and Methods in Physics Research, Section A: Accelerators, Spectrometers, Detectors and Associated Equipment 2011, 633, S243–S246. 10.1016/j.nima.2010.06.178.

[ref43] John J. J.; Brouard M.; Clark A.; Crooks J.; Halford E.; Hill L.; Lee J. W. L.; Nomerotski A.; Pisarczyk R.; Sedgwick I.; et al. PImMS, a fast event-triggered monolithic pixel detector with storage of multiple timestamps. Journal of Instrumentation 2012, 7, C08001 10.1088/1748-0221/7/08/C08001.

[ref44] Garcia G. A.; Nahon L.; Powis I. Two-dimensional charged particle image inversion using a polar basis function expansion. Rev. Sci. Instrum. 2004, 75, 4989–4996. 10.1063/1.1807578.

[ref45] Frasinski L. J.; Codling K.; Hatherly P. A. Covariance Mapping: A Correlation Method Applied to Multiphoton Multiple Ionization. Science 1989, 246, 1029–1031. 10.1126/science.246.4933.1029. 17806394

[ref46] Slater C. S.; Blake S.; Brouard M.; Lauer A.; Vallance C.; John J. J.; Turchetta R.; Nomerotski A.; Christensen L.; Nielsen J. H.; et al. Covariance imaging experiments using a pixel-imaging mass-spectrometry camera. Phys. Rev. A 2014, 89, 011401 10.1103/PhysRevA.89.011401.

[ref47] Cheng C.; Streeter Z. L.; Howard A. J.; Spanner M.; Lucchese R. R.; McCurdy C. W.; Weinacht T.; Bucksbaum P. H.; Forbes R. Strong-field ionization of water. II. Electronic and nuclear dynamics en route to double ionization. Phys. Rev. A 2021, 104, 023108 10.1103/PhysRevA.104.023108.

[ref48] Cockett M. C. R.; Donovan R. J.; Lawley K. P. Zero kinetic energy pulsed field ionization (ZEKE-PFI) spectroscopy of electronically and vibrationally excited states of I_2_^+^: The *A*^2^Π_3/2,*u*_ state and a new electronic state, the *a*^4^Σ_*u*_^–^ state. J. Chem. Phys. 1996, 105, 3347 10.1063/1.472535.

[ref49] de Jong W. A.; Visscher L.; Nieuwpoort W. C. Relativistic and correlated calculations on the ground, excited, and ionized states of iodine. J. Chem. Phys. 1997, 107, 9046 10.1063/1.475194.

[ref50] Zhu J. S.; Deng J. K.; Ning C. G. High-resolution electron-momentum spectroscopy of the valence orbitals of the iodine molecule. Phys. Rev. A 2012, 85, 052714 10.1103/PhysRevA.85.052714.

[ref51] Yencha A. J.; Cockett M. C.; Goode J. G.; Donovan R. J.; Hopkirk A.; King G. C. Threshold photoelectron spectroscopy of I_2_. Chem. Phys. Lett. 1994, 229, 347–352. 10.1016/0009-2614(94)01060-9.

[ref52] Kramida A.; Ralchenko Yu.; Reader J.; NIST ASD Team NIST Atomic Spectra Database (ver. 5.9); National Institute of Standards and Technology: Gaithersburg, MD, 2021; https://physics.nist.gov/asd [2022, June 5].

[ref53] LeRoy R. J. Spectroscopic Reassignment and Ground-State Dissociation Energy of Molecular Iodine. J. Chem. Phys. 1970, 52, 2678–2682. 10.1063/1.1673357.

[ref54] Normand D.; Schmidt M. Multiple ionization of atomic and molecular iodine in strong laser fields. Phys. Rev. A 1996, 53, R1958 10.1103/PhysRevA.53.R1958. 9913224

[ref55] Niikura H.; Légaré F.; Hasbani R.; Bandrauk A.; Ivanov M. Y.; Villeneuve D.; Corkum P. Sub-laser-cycle electron pulses for probing molecular dynamics. Nature 2002, 417, 917–922. 10.1038/nature00787. 12087396

[ref56] Schell F.; Boguslavskiy A. E.; Schulz C. P.; Patchkovskii S.; Vrakking M. J.; Stolow A.; Mikosch J. Sequential and direct ionic excitation in the strong-field ionization of 1-butene molecules. Phys. Chem. Chem. Phys. 2018, 20, 14708–14717. 10.1039/C7CP08195B. 29774327

[ref57] Keldysh L.; et al. Ionization in the field of a strong electromagnetic wave. Sov. Phys. JETP 1965, 20, 1307–1314.

[ref58] Allum F.; Cheng C.; Howard A. J.; Bucksbaum P. H.; Brouard M.; Weinacht T.; Forbes R. Multi-Particle Three-Dimensional Covariance Imaging:“Coincidence” Insights into the Many-Body Fragmentation of Strong-Field Ionized D2O. J. Phys. Chem. Lett. 2021, 12, 8302–8308. 10.1021/acs.jpclett.1c02481. 34428066

[ref59] Slater C. S.; Blake S.; Brouard M.; Lauer A.; Vallance C.; Bohun C. S.; Christensen L.; Nielsen J. H.; Johansson M. P.; Stapelfeldt H. Coulomb-explosion imaging using a pixel-imaging mass-spectrometry camera. Phys. Rev. A 2015, 91, 053424 10.1103/PhysRevA.91.053424.

[ref60] Gibson G. N.; Li M.; Guo C.; Nibarger J. P. Direct evidence of the generality of charge-asymmetric dissociation of molecular iodine ionized by strong laser fields. Phys. Rev. A 1998, 58, 4723–4727. 10.1103/PhysRevA.58.4723.

[ref61] Guo C.; Li M.; Gibson G. N. Charge asymmetric dissociation induced by sequential and nonsequential strong field ionization. Phys. Rev. Lett. 1999, 82, 2492–2495. 10.1103/PhysRevLett.82.2492.

[ref62] Corrales M. E.; Gitzinger G.; González-Vázquez J.; Loriot V.; de Nalda R.; Bañares L. Velocity Map Imaging and Theoretical Study of the Coulomb Explosion of CH_3_I under Intense Femtosecond IR Pulses. J. Phys. Chem. A 2012, 116, 2669–2677. 10.1021/jp207367a. 22103792

[ref63] Allum F.; Anders N.; Brouard M.; Bucksbaum P. H.; Burt M.; Downes-ward B.; Grundmann S.; Harries J.; Ishimura Y.; Iwayama H.; et al. Multi-channel photodissociation and XUV-induced charge transfer dynamics in strong-field-ionized methyl iodide studied with time-resolved recoil-frame covariance imaging. Faraday Discuss. 2021, 228, 571–596. 10.1039/D0FD00115E. 33629700

[ref64] Crane S. W.; Ge L.; Cooper G. A.; Carwithen B. P.; Bain M.; Smith J. A.; Hansen C. S.; Ashfold M. N. Nonadiabatic Coupling Effects in the 800 nm Strong-Field Ionization-Induced Coulomb Explosion of Methyl Iodide Revealed by Multimass Velocity Map Imaging andAb InitioSimulation Studies. J. Phys. Chem. A 2021, 125, 9594–9608. 10.1021/acs.jpca.1c06346. 34709807

[ref65] Hishikawa A.; Liu S.; Iwasaki A.; Yamanouchi K. Light-induced multiple electronic-state coupling of O 2+ in intense laser fields. J. Chem. Phys. 2001, 114, 9856–9862. 10.1063/1.1368383.

[ref66] Alnaser A.; Voss S.; Tong X.-M.; Maharjan C.; Ranitovic P.; Ulrich B.; Osipov T.; Shan B.; Chang Z.; Cocke C. Effects of molecular structure on ion disintegration patterns in ionization of O 2 and N 2 by short laser pulses. Physical review letters 2004, 93, 113003 10.1103/PhysRevLett.93.113003. 15447336

[ref67] Kłoda T.; Matsuda A.; Karlsson H. O.; Elshakre M.; Linusson P.; Eland J. H.; Feifel R.; Hansson T. Strong-field photoionization of O_2_ at intermediate light intensity. Phys. Rev. A 2010, 82, 033431 10.1103/PhysRevA.82.033431.

[ref68] Guo W.; Wang Y.; Li Y. Femtosecond photoelectron imaging of NO at 410nm. Optik 2018, 161, 151–155. 10.1016/j.ijleo.2018.02.019.

[ref69] Wiese J.; Olivieri J.-F.; Trabattoni A.; Trippel S.; Küpper J. Strong-field photoelectron momentum imaging of OCS at finely resolved incident intensities. New J. Phys. 2019, 21, 083011 10.1088/1367-2630/ab34e8.

[ref70] Donovan R. J.; Flood R. V.; Lawley K. P.; Yencha A. J.; Ridley T. The resonance enhanced (2 + 1) multiphoton ionization spectrum of I_2_. Chem. Phys. 1992, 164, 439–450. 10.1016/0301-0104(92)87080-S.

[ref71] Donovan R. J.; Flexen A. C.; Lawley K. P.; Ridley T. The (2 + 1) REMPI spectroscopy of jet-cooled Br_2_. Chem. Phys. 1998, 226, 217–228. 10.1016/S0301-0104(97)00324-8.

[ref72] Allum F.; Music V.; Inhester L.; Boll R.; Erk B.; Schmidt P.; Baumann T. M.; Brenner G.; Burt M.; Demekhin P. V.; et al. A localized view on molecular dissociation via electron-ion partial covariance. Communications Chemistry 2022, 5, 1–10. 10.1038/s42004-022-00656-w. PMC9814695 36697752

[ref73] de Boer M. P.; Muller H. G. Observation of large populations in excited states after short-pulse multiphoton ionization. Phys. Rev. Lett. 1992, 68, 2747 10.1103/PhysRevLett.68.2747. 10045482

[ref74] Jones R. R.; Schumacher D. W.; Bucksbaum P. H. Population trapping in Kr and Xe in intense laser fields. Phys. Rev. A 1993, 47, R49–R52. 10.1103/PhysRevA.47.R49. 9908994

[ref75] Manschwetus B.; Nubbemeyer T.; Gorling K.; Steinmeyer G.; Eichmann U.; Rottke H.; Sandner W. Strong laser field fragmentation of H_2_: Coulomb explosion without double ionization. Phys. Rev. Lett. 2009, 102, 113002 10.1103/PhysRevLett.102.113002. 19392198

[ref76] Ma J.; Zhang W.; Lin K.; Ji Q.; Li H.; Sun F.; Qiang J.; Chen F.; Tong J.; Lu P.; et al. Strong-field dissociative Rydberg excitation of oxygen molecules: Electron-nuclear correlation. Phys. Rev. A 2019, 100, 063413 10.1103/PhysRevA.100.063413.

[ref77] Zhang W.; Lu P.; Ma J.; Li H.; Gong X.; Wu J. Correlated electron–nuclear dynamics of molecules in strong laser fields. J. Phys. B At. Mol. Opt. Phys. 2020, 53, 162001 10.1088/1361-6455/ab9763.

[ref78] Kvaran A.; Yencha A. J.; Kela D. K.; Donovan R. J.; Hopkirk A. Vibrationally resolved excitation functions for direct ion-pair (I++ I-) formation from photodissociation of I2. Chemical physics letters 1991, 179, 263–267. 10.1016/0009-2614(91)87035-A.

[ref79] Lawley K.; Ridley T.; Min Z.; Wilson P.; Al-Kahali M.; Donovan R. Vibronic coupling between Rydberg and ion-pair states of I2 investigated by (2+ 1) resonance enhanced multiphoton ionization spectroscopy. Chemical physics 1995, 197, 37–50. 10.1016/0301-0104(95)00158-K.

[ref80] Hanstorp D.; Gustafsson M. Determination of the electron affinity of iodine. Journal of Physics B: Atomic, Molecular and Optical Physics 1992, 25, 1773–1783. 10.1088/0953-4075/25/8/012.

[ref81] Bhardwaj V. R.; Mathur D.; Rajgara F. A. Formation of Negative Ions upon Irradiation of Molecules by Intense Laser Fields. Phys. Rev. Lett. 1998, 80, 3220 10.1103/PhysRevLett.80.3220.

