# Supplementary material for: Photoionization and Photofragmentation Dynamics of I2 in Intense Laser Fields: A Velocity-Map Imaging Study
Source: J Phys Chem A. 2022 Nov 9;126(46):8577–87. doi: 10.1021/acs.jpca.2c04379 (PMC9706571; doi:10.1021/acs.jpca.2c04379)
Supplement: Supplementary file 1 — jp2c04379_si_002.pdf [file jp2c04379_si_002.pdf]

Supporting Information for: The photoionization and photofragmentation dynamics of I_2 in intense laser fields: a velocity-map imaging study

Felix Allum,[*,†,‡] Joseph McManus,[†] Oskar Denby,[†] Michael Burt,[†] and Mark Brouard[*,†]

†*Chemistry Research Laboratory, Department of Chemistry, University of Oxford, Oxford OX1 3TA, United Kingdom*

‡*Current Address: PULSE Institute, SLAC National Accelerator Laboratory, 2575 Sand Hill Road, Menlo Park, CA 94025, USA*

E-mail: fallum@stanford.edu; mark.brouard@chem.ox.ac.uk

Comparison of I^+ Ion Image To Literature

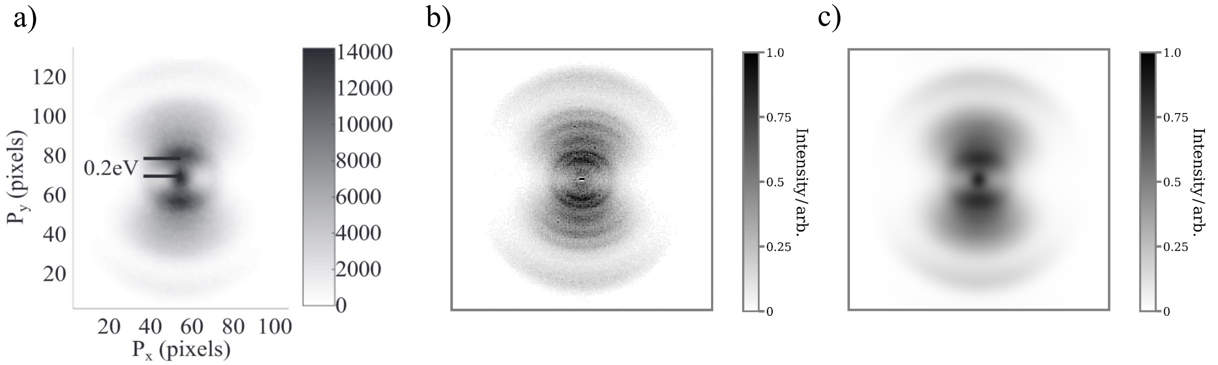

Figure S1: Comparison between the I^+ ion image reported in the current work with that previously reported in literature.[1] a) I^+ Image reported in reference 1. b) I^+ image from the current work. c) I^+ image from the current work convoluted by a 2D Gaussian function with standard deviation of five pixels. For panels b) and c), the colourmap and radial scale has been chosen to allow direct comparison to the literature image. Reprinted (Adapted or Reprinted in part) with permission from Reference 1. Copyright 2017, APS.

As discussed in the main text, there has been a recent velocity-map imaging study on I_2 in intense laser fields at a range of laser wavelengths, including 800 nm.[1] In the work of Smith *et al.*, fewer I_2^+ photodissociation channels following 800 nm ionization could be distinguished in the I^+ ion image than in the current work. Comparison of the VMI images shown in the current work and in the study of Smith *et al.* (Figure S1 panels a) and b)) suggest that this is primarily due to the difference in the achieved velocity resolution. Indications for an improved velocity resolution in the current work can be seen in the raw images, with, for instance, the I_2^{2+} feature at zero radius appearing much sharper in panel b) than panel a). Convolution of our I^+ image with a Gaussian function in x and y with a standard deviation of 5 pixels, to simulate a degradation of velocity resolution, produces rather strong agreement to the literature image, as demonstrated in Figure S1c).

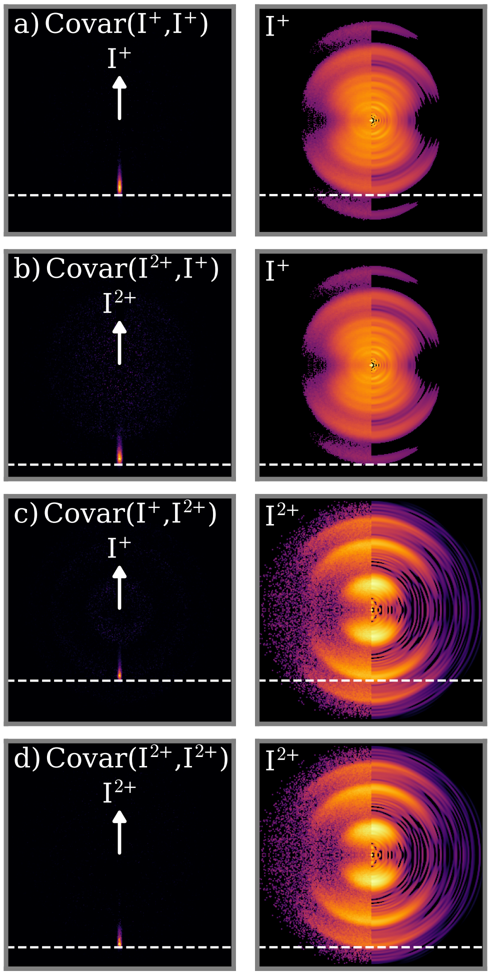

Figure S2: Ion covariance imaging analysis. Panels a) to d) show the recoil-frame covariance image for different pairs of ions (left), which are compared to the raw ion image for the plotted ion (right). The recoil-frame covariance images are labelled 'Covar(A^+,B^+)' for the combination of a reference ion A^+ and a plotted ion B^+. Horizontal dashed white lines drawn across both images indicate the approximate position of the feature in the recoil-frame covariance image. The VMI images are plotted using a logarithmic colour scale as in Figure 4a) of the main manuscript. In the case where covariance is calculated between an ion and itself, the autovariance signal (at a recoil angle of 0 degrees) has been masked.

Ion Recoil-Frame Covariance Imaging Analysis

The use of ion-ion covariance imaging analysis can support some of the assessments made about the ion imaging data in the main text. By calculating the covariance between the event-based ion imaging data produced in a multi-mass VMI experiment with a timestamping detector, information can be gained about correlations between features in the recorded ion images, and thus the photodissociation/Coulomb explosion channels themselves. A detailed description of the covariance analysis procedure used can be found in references 2–4. In short, the covariance between the intensities between each pair of pixels between two ion images is calculated. The covariance is then plotted in a frame such that one ion (termed the 'reference') has its velocity constrained to a particular direction, and the velocity distribution of the second ion (the 'plotted') is shown relative to this.

Figure S2 displays calculated ion-ion covariance images for a series of combinations of reference and plotted ions. In each case, these are compared to the raw ion image associated with the plotted ion. In each pair of ions, a sharp signal at 180 degrees recoil angle is observed, indicative of a two body Coulomb explosion. By comparing the radius of this feature to that in the raw ion image, assignments of specific Coulomb explosion channels can be made. In Figure S2 this comparison is made by overlaying a horizontal white dashed line at the approximate position of the back-to-back feature in the recoil-frame covariance feature, which can be seen to line up with a specific ring in the VMI image. For instance, in panel a), the feature seen in the (I^+,I^+) covariance image occurs at the same radius as the feature previously assigned to (1,1) Coulomb explosion, as expected. Additionally, features in the ion images which are not present in any of the covariance images can be assigned to dissociations in which only one charged species is produced (and thus, is responsible for no correlations between pairs of ions). This is the case for the lower radius features in the I^+ and I^{2+} VMI images, arising from (0,1) and (0,2) dissociations of the parent cation and dication, respectively.

Predicted Dissociation KERs

In Figure 2 of the main text, a series of predicted KERs are shown for dissociation channels assuming different combinations of known electronic states of the I_2^+ cation and electronic states of the $I+I^+$ dissociation products. For this, vertical excitation was assumed, using Equation (1) in the main text. Vertical ionization energies were taken from reference 6. Asymptotic energies of the dissociation products were taken from reference 7. Details of the dissociation limits and cation states considered, as well as the numerical values of the predicted KERs are listed in Table S1.

	Dissociation limit (I+I$^+$)						
Ion State (I_v)	D1 ($^2P_{3/2}$+$^2P_2$)	D2 ($^2P_{3/2}$+$^2P_{0,1}$)	D3 ($^2P_{1/2}$+$^2P_{0,1}$)	D4 ($^2P_{1/2}$+$^1D_2$)	D5 ($^2P_{3/2}$+^1S)	D6 ($^2P_{1/2}$+^1S)	D7 (($^2[2]_{5/2,3/2}$+$^2P_2$)
A (13.90)	1.91	1.04	0.15	-	-	-	-
B (16.88)	-	-	3.13	2.25	1.23	0.29	-
C_1 (19.36)	-	-	-	-	3.71	2.77	0.51
C_2 (20.21)	-	-	-	-	-	3.62	1.36
D (21.53)	-	-	-	-	-	-	2.68

Table S1: Predicted KERs for different possible I_2^+ dissociation channels. Only KERs of less than 4 eV are shown. The dissociation limits are labelled in the same manner as references 5 and 1, and the electronic configuration of the neutral and iodine product in each dissociation limit is given. The labelling of ion states and dissociation limits is the same as that used in Figure 2 of the main manuscript.

Ion Mass Spectrum

Figure S3 shows an example ion mass spectrum recorded at a peak laser intensity of 35 TWcm^{-2}. This data was recorded by defocussing the ion optics voltages by changing the repeller to extractor voltage ratio significantly from the optimum for velocity-mapping. This avoids saturation of the zero KE parent ion signal (I_2^+).

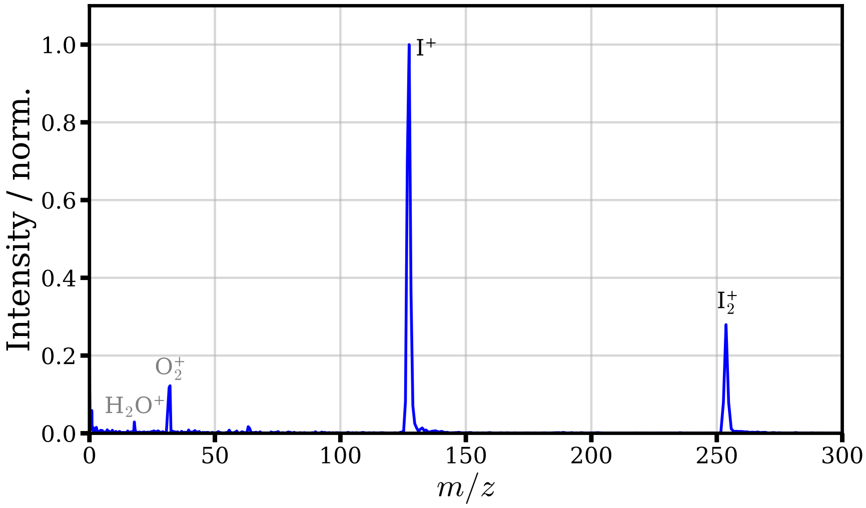

Figure S3: Example ion mass spectrum recorded at a peak intensity of 35 TWcm^{-2}.

Intensity-dependent ion imaging

Figure S4 displays I^+ KER distributions as a function of peak laser intensity, focussing solely on the low KER region associated with I_2^+ dissociation (in contrast to Figure 3 of the main manuscript, which shows an expanded KER range, including signals from dication and trication dissociations). Figure S5 displays extracted peak centres and amplitudes for a fit of these KER distributions to a series of Gaussian contributions, as discussed in the main text. It can be seen that the centres of the peaks do not vary with laser intensity. In contrast, the amplitudes of Channels I and II decrease significantly as laser intensity is increased, with concordant increases in the amplitudes of the higher KER channels.

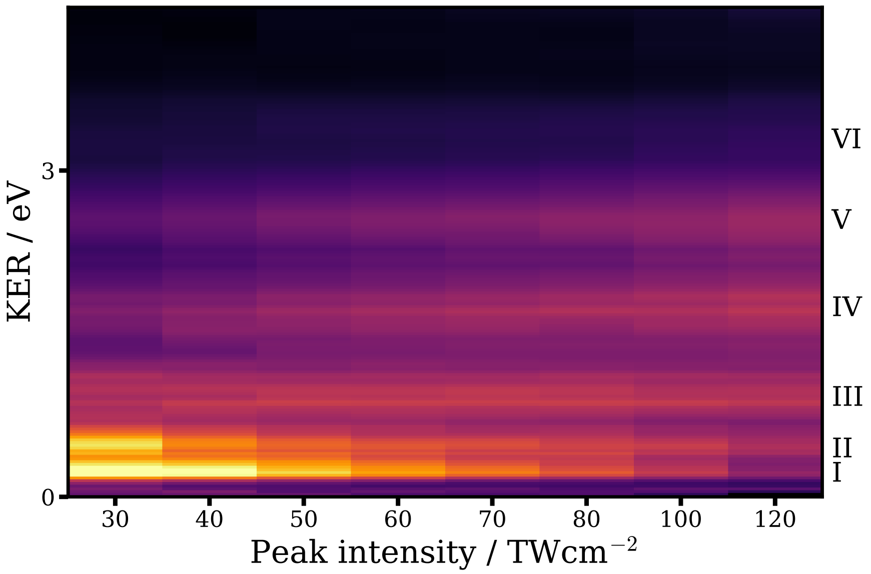

Figure S4: The KER distributions (normalized to unit total intensity) extracted from a series of I^+ VMI images as a function of peak laser intensity. The different I_2^+ dissociation channels discussed in the main text are labelled. This figure displays the same data as Figure 3 of the main text, but focusses on the lower KER region associated with monocation dissociation.

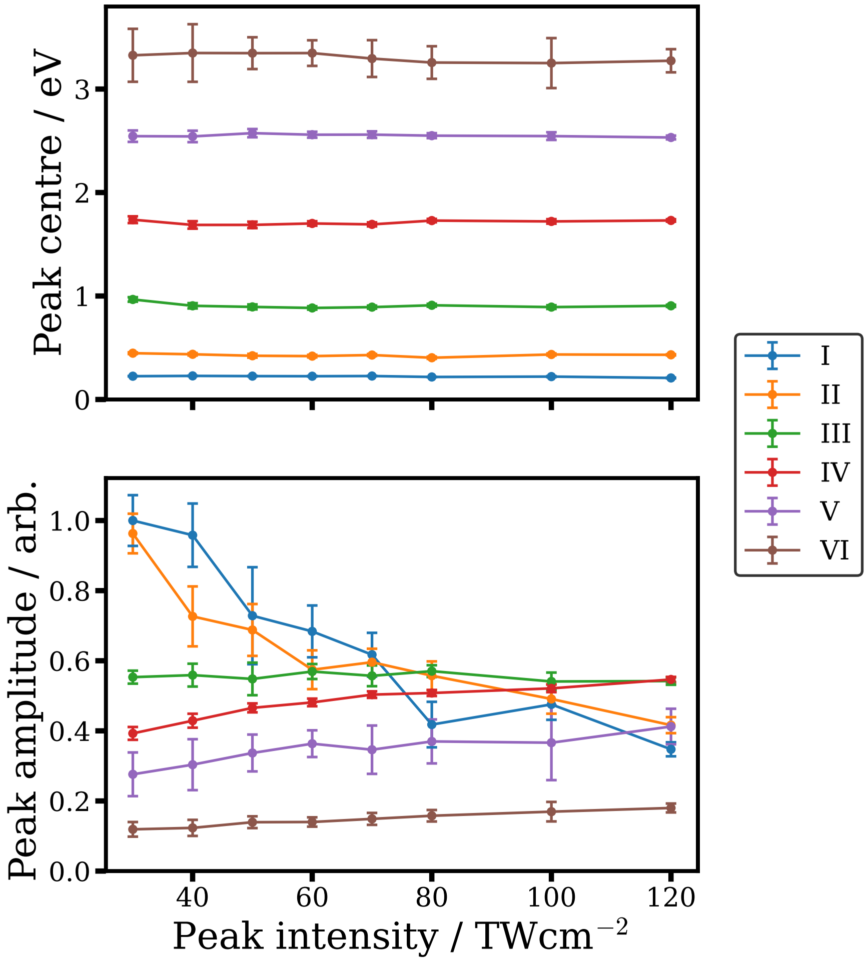

Figure S5: Extracted quantities (top: Gaussian peak centre, bottom: Gaussian peak amplitude) from fitting the intensity-dependent I^+ KER distributions to a series of Gaussian contributions, as discussed in the main text.

Intensity-dependent anion imaging

Figure S6 shows ratio of total anion (I^-) to electron signal recorded as a function of peak laser intensity. This ratio decreases as laser intensity increases, which is consistent with stronger fields increasing the probability of photodetatchement of nascent I^- ions, or of photoionization of the precursor highly excited neutral Rydberg states.

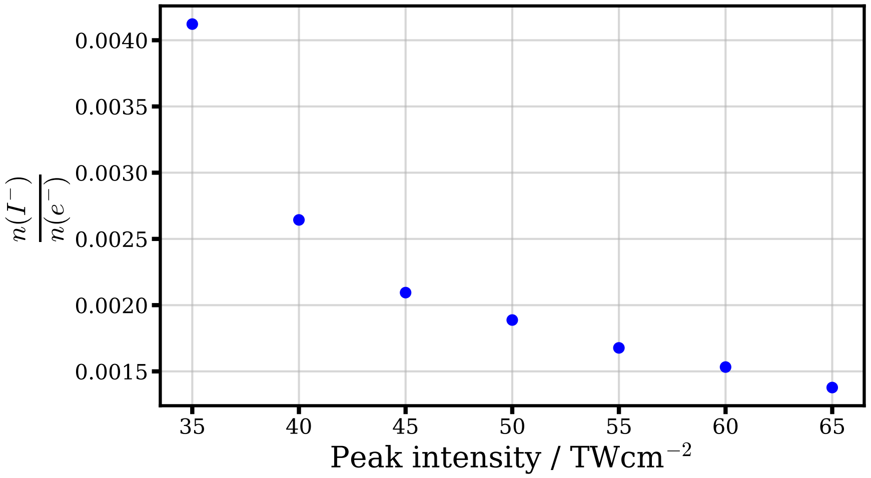

Figure S6: The ratio of total I^- signal to total electron signal recorded as a function of peak laser intensity.

References

(1) Smith, D. L.; Tagliamonti, V.; Dragan, J.; Gibson, G. N. Single ionization of molecular iodine. *Physical Review A* **2017**, *95*, 1–8.

(2) Slater, C. S.; Blake, S.; Brouard, M.; Lauer, A.; Vallance, C.; John, J. J.; Turchetta, R.; Nomerotski, A.; Christensen, L.; Nielsen, J. H. et al. Covariance imaging experiments using a pixel-imaging mass-spectrometry camera. *Phys. Rev. A* **2014**, *89*, 011401.

(3) Slater, C. S.; Blake, S.; Brouard, M.; Lauer, A.; Vallance, C.; Bohun, C. S.; Christensen, L.; Nielsen, J. H.; Johansson, M. P.; Stapelfeldt, H. Coulomb-explosion imaging using a pixel-imaging mass-spectrometry camera. *Physical Review A* **2015**, *91*, 053424.

(4) Allum, F.; Anders, N.; Brouard, M.; Bucksbaum, P. H.; Burt, M.; Downes-ward, B.; Grundmann, S.; Harries, J.; Ishimura, Y.; Iwayama, H. et al. Multi-channel photodissociation and XUV-induced charge transfer dynamics in strong-field-ionized methyl iodide studied with time-resolved recoil-frame covariance imaging. *Faraday Discuss.* **2021**, *228*, 571–596.

(5) de Jong, W. A.; Visscher, L.; Nieuwpoort, W. C. Relativistic and correlated calculations on the ground, excited, and ionized states of iodine. *J. Chem. Phys.* **1997**, *107*, 9046.

(6) Zhu, J. S.; Deng, J. K.; Ning, C. G. High-resolution electron-momentum spectroscopy of the valence orbitals of the iodine molecule. *Phys. Rev. A* **2012**, *85*, 052714.

(7) Kramida, A.; Yu. Ralchenko,; Reader, J.; and NIST ASD Team, NIST Atomic Spectra Database (ver. 5.9), [Online]. Available: `https://physics.nist.gov/asd` [2022, June 5]. National Institute of Standards and Technology, Gaithersburg, MD., 2021.
